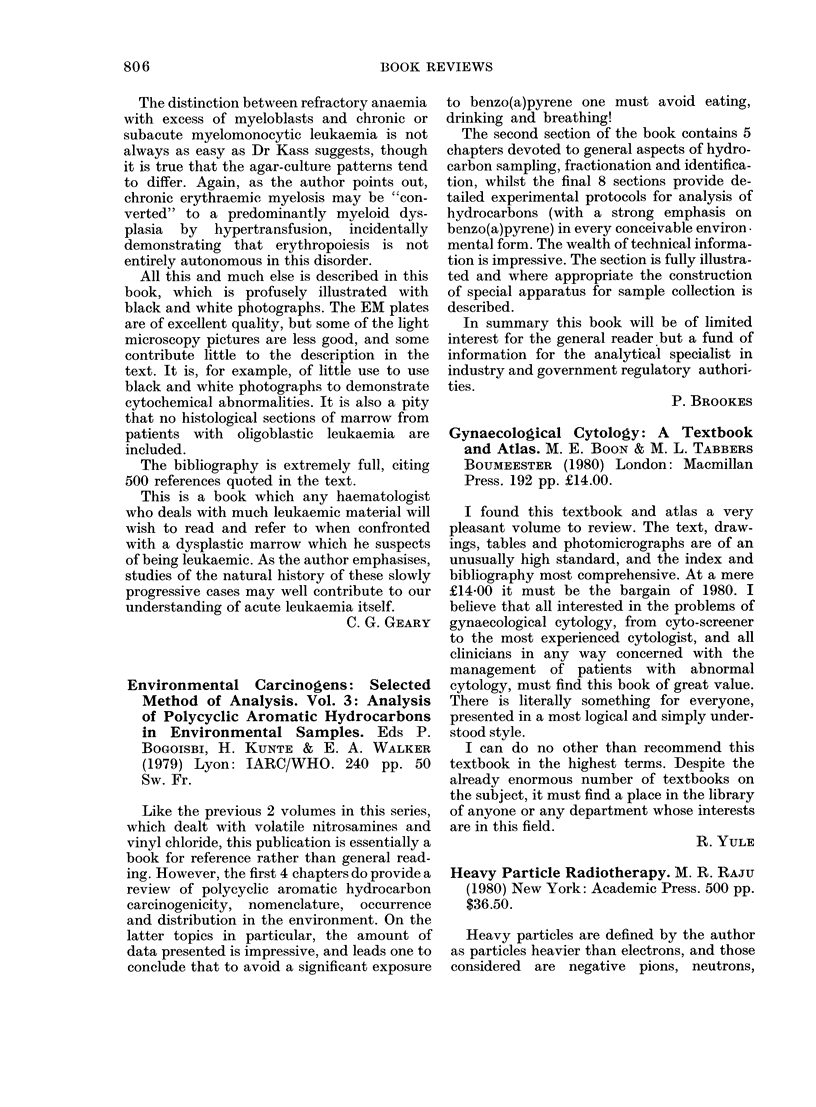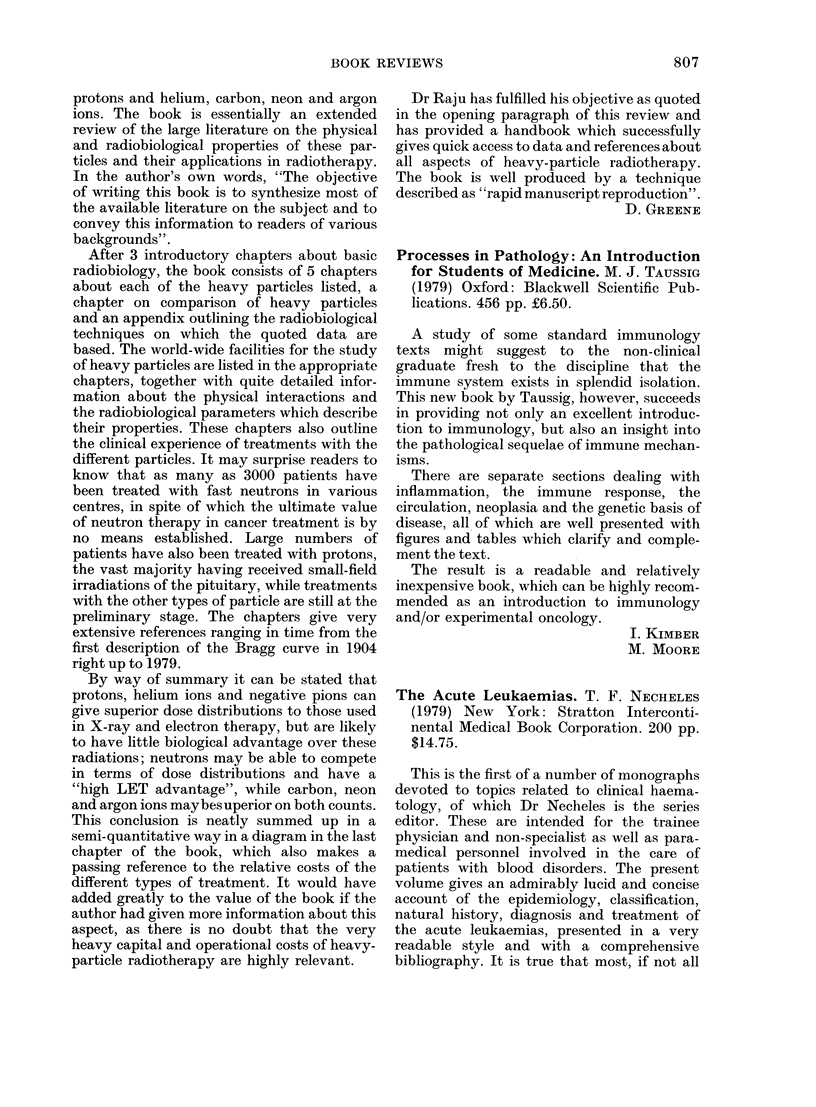# Heavy Particle Radiotherapy

**Published:** 1980-11

**Authors:** D. Greene


**Heavy Particle Radiotherapy.** M. R. Raju (1980) New York: Academic Press. 500 pp. $36.50.

Heavy particles are defined by the author as particles heavier than electrons, and those considered are negative pions, neutrons, protons and helium, carbon, neon and argon ions. The book is essentially an extended review of the large literature on the physical and radiobiological properties of these particles and their applications in radiotherapy. In the author's own words, "The objective of writing this book is to synthesize most of the available literature on the subject and to convey this information to readers of various backgrounds".

After 3 introductory chapters about basic radiobiology, the book consists of 5 chapters about each of the heavy particles listed, a chapter on comparison of heavy particles and an appendix outlining the radiobiological techniques on which the quoted data are based. The world-wide facilities for the study of heavy particles are listed in the appropriate chapters, together with quite detailed information about the physical interactions and the radiobiological parameters which describe their properties. These chapters also outline the clinical experience of treatments with the different particles. It may surprise readers to know that as many as 3000 patients have been treated with fast neutrons in various centres, in spite of which the ultimate value of neutron therapy in cancer treatment is by no means established. Large numbers of patients have also been treated with protons, the vast majority having received small-field irradiations of the pituitary, while treatments with the other types of particle are still at the preliminary stage. The chapters give very extensive references ranging in time from the first description of the Bragg curve in 1904 right up to 1979.

By way of summary it can be stated that protons, helium ions and negative pions can give superior dose distributions to those used in X-ray and electron therapy, but are likely to have little biological advantage over these radiations; neutrons may be able to compete in terms of dose distributions and have a "high LET advantage", while carbon, neon and argon ions maybesuperior on both counts. This conclusion is neatly summed up in a semi-quantitative way in a diagram in the last chapter of the book, which also makes a passing reference to the relative costs of the different types of treatment. It would have added greatly to the value of the book if the author had given more information about this aspect, as there is no doubt that the very heavy capital and operational costs of heavy-particle radiotherapy are highly relevant.

Dr Raju has fulfilled his objective as quoted in the opening paragraph of this review and has provided a handbook which successfully gives quick access to data and references about all aspects of heavy-particle radiotherapy. The book is well produced by a technique described as "rapid manuscript reproduction".

D. Greene